# Clinical improvement of protein-losing enteropathy after SGLT2 inhibitor therapy in an adult with failing Fontan circulation: a case report

**DOI:** 10.1093/ehjcr/ytag117

**Published:** 2026-02-17

**Authors:** Emanuela C D’Angelo, Veronica Bordonaro, Rosalinda Palmieri, Micol Rebonato, Claudia Montanaro

**Affiliations:** Adult Congenital Heart Disease Unit, Bambino Gesù Children’s Hospital, IRCCS, Piazza di Sant'Onofrio, 4, 00165 Roma RM, Italy; Guy's and St Thomas' NHS Foundation Trust, Westminster Bridge Road, London SE1 7EH, UK; Advanced Cardiothoracovascular and Fetal Imaging Unit, Bambino Gesù Children’s Hospital, IRCCS, Piazza di Sant'Onofrio, 4, 00165 Roma RM, Italy; Adult Congenital Heart Disease Unit, Bambino Gesù Children’s Hospital, IRCCS, Piazza di Sant'Onofrio, 4, 00165 Roma RM, Italy; Interventional Cardiology Unit, Bambino Gesù Children’s Hospital, IRCCS, Piazza di Sant'Onofrio, 4, 00165 Roma RM, Italy; Adult Congenital Heart Disease Unit, Bambino Gesù Children’s Hospital, IRCCS, Piazza di Sant'Onofrio, 4, 00165 Roma RM, Italy; National Heart and Lung Institute, Imperial College London, South Kensington Campus, London SW7 2AZ, UK

**Keywords:** Adult congenital heart disease, Fontan circulation, Heart failure, Sodium–glucose cotransporter 2 inhibitors, Case report

## Abstract

**Background:**

Sodium–glucose cotransporter 2 inhibitors (SGLT-2i) have proven benefits in patients with biventricular heart failure; however, their role in patients with univentricular physiology and failing Fontan circulation (FC) remains largely unexplored.

**Clinical Presentation:**

A 21-year-old male with failing FC, preserved ejection fraction, and protein-loosing enteropathy (PLE) presented to our Adult Congenital Heart Disease Unit for evaluation. Born with a L-transposition of the great vessels with an interventricular septal defect and antero-superior rudimentary chamber, he underwent a Fontan completion at age 5. Since he was 16 years old, he had multiple admissions for FC failure complicated by PLE and ascites, which did not respond to conventional treatments including diuretics, corticosteroids, albumin, and immunoglobulin infusions. Despite the stenosis of the extracardiac conduit, treated by the placement of a covered stent and a further conduit balloon dilation and stenting in a stent procedure performed after 3 years, the patient continued to experience ascites and hypoalbuminemia. To better understand the extent of his lymphatic dysfunction, magnetic resonance lymphoscintigraphy was performed. The study showed significant lymphatic congestion, with leakage into the duodenal lumen and prominent stasis in the thoracic duct. Based on these findings, therapy with SGLT-2i was initiated. Over the following 18 months, there was marked clinical improvement with a reduction in ascites and stabilization of serum protein and albumin levels.

**Conclusions:**

This case highlights the potential benefit of SGLT-2i in managing Fontan failure complicated by PLE. Multicenter studies are needed to further investigate the efficacy, safety, and mechanism of action of SGLT-2i in this unique patient population.

Learning points

- SGLT2 inhibitors in this case might have served as an adjunctive therapy in managing Fontan failure complicated by PLE, together with conduit stenting optimization.
- Lymphatic imaging, including lymphatic MRI, is a valuable diagnostic tool to identify lymphatic congestion and leakage, to possibly guide personalized treatment strategies in patients with failing of the Fontan circulation.

## Introduction

Sodium-glucose cotransporter 2 inhibitors (SGLT-2i) reduce the composite end-point of worsening heart failure (HF) and cardiovascular death in patients with biventricular physiology in the presence of HF with reduced or preserved ejection fraction (EF).^1,2^ Their effect in patients with univentricular physiology and failing Fontan circulation (FC) is not yet clearly established.^3–5^ The aim of this case report is to illustrate the potential therapeutic effect of SGLT-2i in a patient with failing FC and protein-losing enteropathy (PLE).

## Summary figure

**Figure:**
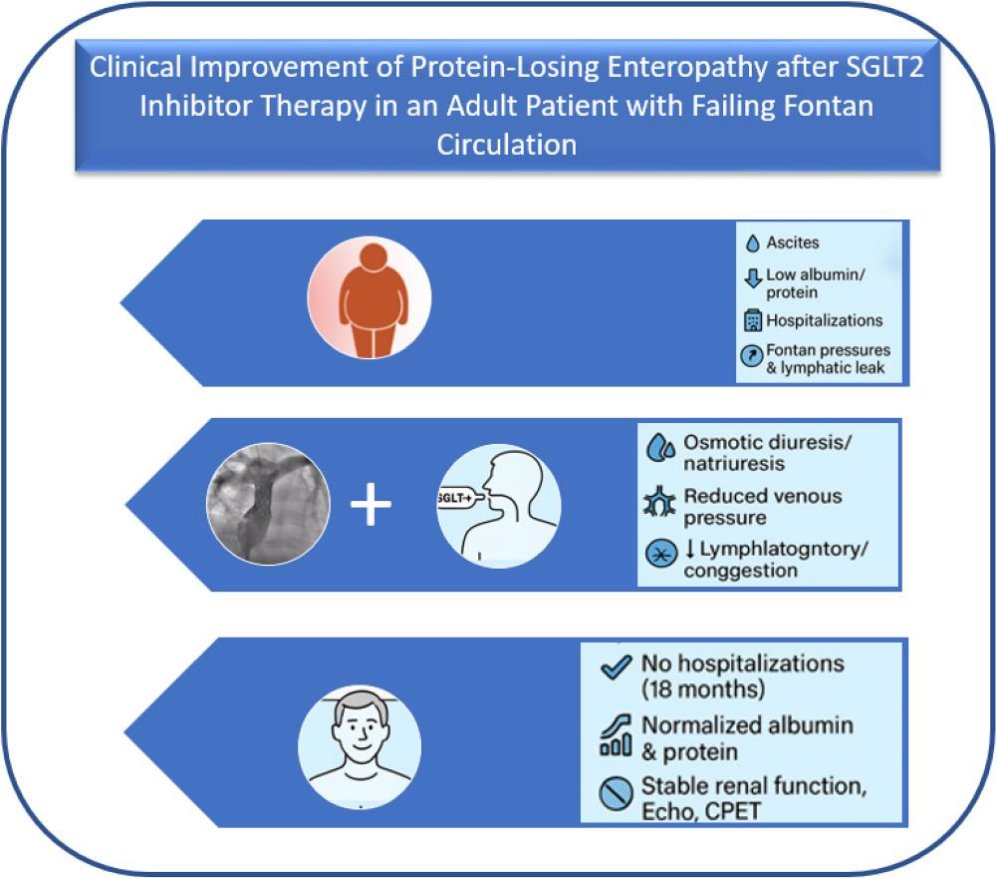


## Case presentation

A 21-years-old male with failing Fontan circulation (FC), preserved ejection fraction of the single ventricle, and protein-losing enteropathy (PLE) was followed in our Adult Congenital Heart Disease (ACHD) Unit.

He was born with L-transposition of the great arteries with an interventricular septal defect and an antero-superior rudimentary chamber from which the aorta arises. At 20 days of life, he underwent pulmonary artery banding; at 3 years of age, the bandage was removed and he was palliated with a Glenn operation; when he was 5 years old, a Fontan completion was performed with a non-fenestrated extracardiac conduit (Dacron 18 mm).

**Figure 1:**
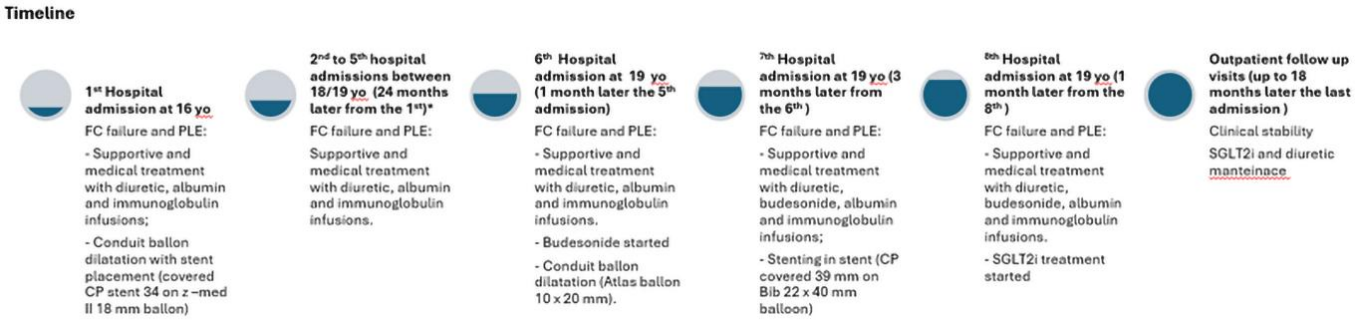
The timeline explains the hospital admissions and the treatment performed. Total of four hospital admissions (second to fifth) between 18/19 years old (24 months later from the first): second admission after 24 months from the first one, third admission after 5 months from the second one, fourth admission 3 months later from the third one, fifth admission 2 months later the fourth one.

Since the age of 16, he experienced several hospital admissions over a 3-year period for FC failure complicated by ascites and PLE (*Figure 1*). Laboratory tests revealed hypoproteinaemia and hypoalbuminemia (*Table 1*); oxygen saturation remained stable at 96%. During the initial admissions, he received supportive therapy with diuretics, albumin, and immunoglobulin infusions.

**Table 1:** Albumin and protein values during hospitalizations and the outpatient clinic follow-up over the following 18 months

	Albumin (g/dL)	Normal Range (g/dL)	Total Proteins (g/dL)	Normal Range (g/dL)
**Hospitalization**				
First	2.4	3.2–4.5	4.4	6.4–8.3
Second	2.4	3.2–4.5	3.8	6.4–8.3
Third	2.7	3.2–4.5	4.1	6.4–8.3
Fourth	2.8	3.4–5	5	6.6–8.7
Fifth	2.2	3.4–5	3.8	6.6–8.7
Sixth	2.1	3.2–4.5	3.4	6.4–8.3
Seventh	2.6	3.4–5	3.9	6.6–8.7
Eighth	2.5	3.4–5	3.8	6.6–8.7
**Outpatient clinics follow-up**				
6 months	4.2	3.4–5	6.8	6.6–8.7
12 months	4.2	3.4–5	6.4	6.6–8.7
18 months	3.7	3.2–4.5	5.4	6.6–8.7

Cardiac catheterization demonstrated the following pressures: Inferior vena cava (IVC) 16 mmHg, superior vena cava (SVC) 14 mmHg, left and right pulmonary arteries (LPA and RPA) 14 mmHg, and pulmonary capillary wedge pressure (PCWP) 10 mmHg. IVC angiography revealed a calcified extracardiac conduit with luminal narrowing (9 × 14 mm in the distal segment), which was successfully treated with the placement of a covered stent.

However, 24 months later, he experienced four additional hospital admissions due to FC failure and PLE and was managed with medical and supportive therapy, including diuretics, albumin, and immunoglobulin infusions.

During the sixth admission for PLE and ascites, the patient underwent a second catheterization, which showed an IVC pressure of 15 mmHg, SVC, LPA, and RPA pressures of 13 mmHg, and a PCWP of 8 mmHg. IVC angiography demonstrated a good result of the previous stenting procedure. Further balloon dilation to remove the 2 mmHg gradient in the pathway was performed. At that point, budesonide was added to the medical therapy.

A few months later, the patient was admitted for a seventh hospitalization. During this admission, another catheterization was performed. Pressures detected were IVC 20 mmHg, SVC, LPA, and RPA 19 mmHg, and PCWP 16 mmHg and a stent-in-stent procedure was performed, achieving a size of 17 × 18 mm.

**Figure 2:**
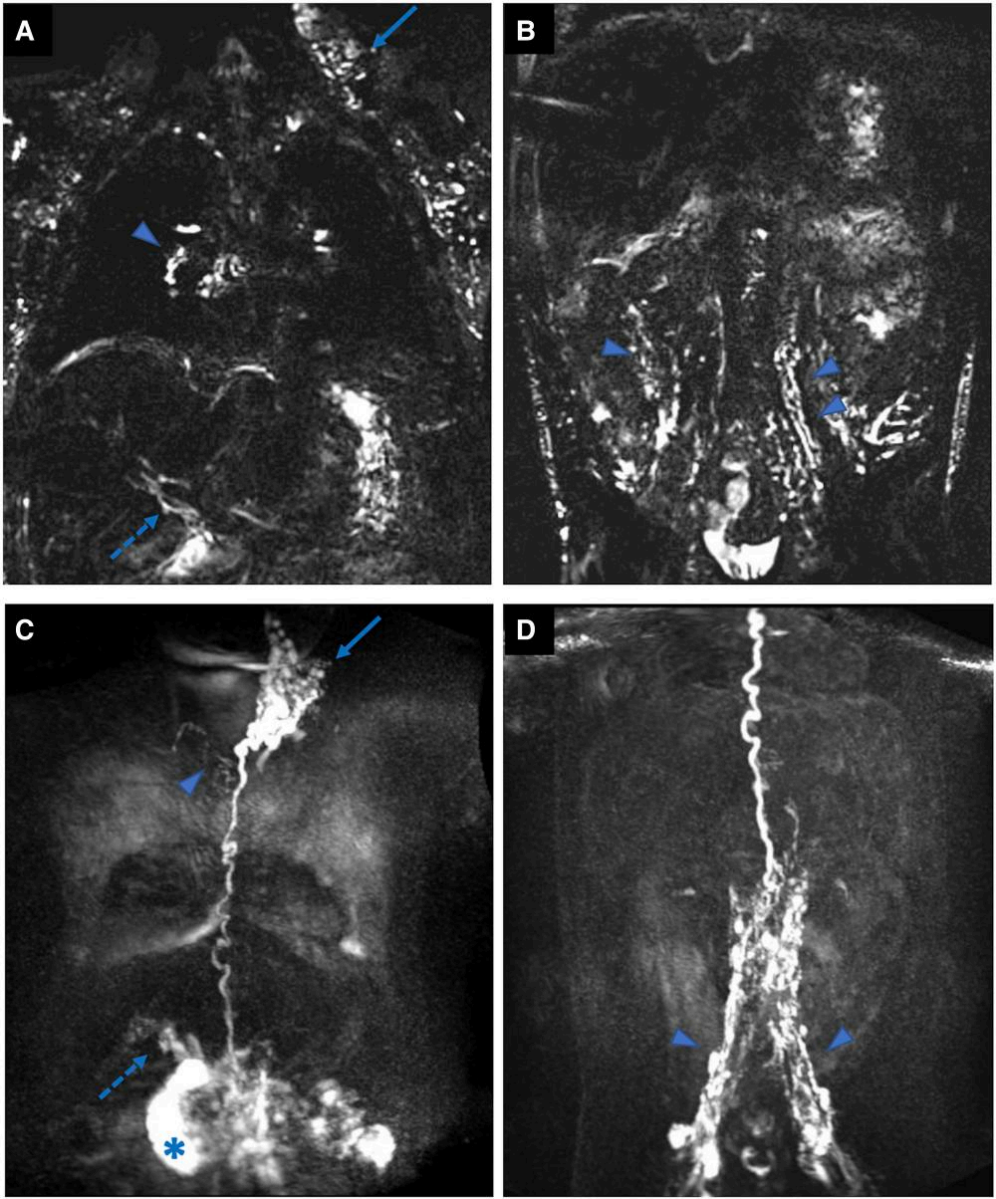
Static 3D-T2 SPACE MR lymphangiography (panels *A* and *B*) shows supradiaphragmatic lymphatic congestion, predominantly in bilateral supraclavicular and laterocervical regions, more evident on the left (arrow in *A*), and in peribronchial mediastinal areas (arrowhead in *A*). Subdiaphragmatic lymphostasis is observed in the periportal and periduodenal regions (dotted arrow in *A*), with mild mesenteric involvement (arrowheads in *B*) and small ascitic fluid collections in the subhepatic and perisplenic spaces, partially loculated in the right paraduodenal area. Dynamic contrast-enhanced MR lymphangiography via transhepatic access (panel *C*) demonstrates progressive opacification of periportal and periduodenal lymphatics (dotted arrow in *C*), with stasis and lymphatic lacunae at the mesenteric root, followed by contrast leakage into the duodenum (*in *C*) and progression into proximal jejunal loops. A single, tortuous thoracic duct with left jugulo-subclavian drainage shows contrast stasis and retrograde flow into left supraclavicular and laterocervical channels (arrow in *C*). A delayed opacification of an accessory subtle channel draining into the right jugulo-subclavian confluence is also evident (arrowhead in *C*). Dynamic contrast-enhanced MR lymphangiography images following inguinal intranodal contrast administration (panel *D*) shows regular retroperitoneal lymphatic drainage, with diffuse lymphatic engorgement along the iliac chains (arrowheads in *D*), with progressive opacification of the single tortuous thoracic duct draining into the left jugulo-subclavian junction.

One month after stenting in stent, the patient was admitted again with hypoproteinemia, hypoalbuminemia, and ascites. A magnetic resonance (MR) lymphangiography study was performed with the intention of planning a percutaneous selected lymphatic occlusion procedure. The static MR lymphangiography showed signs of lymphatic congestion both in the supradiaphragmatic and in the subdiaphragmatic district, and associated ascitic fluid collections in the subhepatic and peri-splenic areas. The dynamic lymphangiography demonstrated a progressive opacification of the subdiaphragmatic district with marked congestion at the mesenteric root and a lymphatic leakage into the duodenal lumen, with progressive intraluminal filling extending to the proximal jejunal loops on the left. Progressive opacification of a single, markedly tortuous thoracic duct was observed within the thoracic cavity, ending at the left jugulo-subclavian junction, with significant stasis and reflux phenomena in the ipsilateral supraclavicular and lateral cervical regions (*Figure 2*).

Therapy with SGLT-2i (Dapagliflozin 10 mg daily) was then initiated, and during the following months the patient showed a marked reduction of the ascites, normalization of protein and albumin levels, which have remained within the normal range up to the most recent follow-up, 18 months after SGLT2i introduction (*Table 1*). Other observations, following the stent procedure and the SGLT2i initiation, were an improved stroke volume and the systolic/diastolic ratio normalization at the follow-up echocardiogram. No deterioration in echocardiograms and in cardiopulmonary exercise test (CPET) parameters was detected. Neither side effects nor renal function decline were noted. (*Table 2*). The patient remained free from hospitalization over an 18-month follow-up period. The interventional lymphatic occlusion procedure has not been performed until now, given the clinical improvement.

**Table 2:** Comparison of laboratory data, echocardiographic findings, and CPET results before and after SGLT2i treatment and stent-in-stent implantation (before 4 and 3 months and after 18 and 19 months, respectively)

	Pre-SGLT2i and stent-in-stent	Post-SGLT2i and stent in stent
Bloods		
Albumin (g/dL)	2.1 g/dL	3.7 g/dL
Total protein (g/dL)	3.4 g/dL	5.4 g/dL
HB (g/dL)	16.7 g/dL	16.9 g/dL
NT-proBNP (pg/mL)	69.6 pg/mL	39 pg/mL
Creatinine (mg/dL)	0.81 mg/dL	0.75 mg/dL
GFR (mL/kg/min)	130 mL/min	133 mL/min
Echocardiogram		
LVDV (mL)	96 mL	133 mL
LVSV (mL)	42 mL	49 mL
EF (%)	56%	64%
S/D ratio	1.1	0.9
SV (mL)	54 mL	84 mL
CPET		
VO_2_ peak (mL/kg/min)	19 mL/kg/min	22 mL/kg/min
Predicted VO_2_ peak (%)^a^	40%	41%
VO_2_/WR (mL/min/watt)	8.5 mL/min/watt	missing
VE/VCO_2_ peak	39.4	39
Pulse of oxygen (mL/beat)	8.2 mL/beat	missing

Abbreviations: LVDV, left ventricular diastolic volume; LVSV, left ventricular systolic volume; NT-proBNP, NT-pro brain natriuretic peptide; S/D ratio, systole/diastole ratio; SV, stroke volume; VO_2_, volume of oxygen consumption.

^a^Predicted with Wasserman tables.

## Discussion

This case highlights the positive effects of SGLT-2i in an adult patient with failing FC and PLE despite a preserved single-ventricle EF. Whilst SGLT-2i are well-established medications in patients with biventricular heart failure,^1,2^ their role and safety in failing univentricular physiology remains largely unexplored.^3–5^

FC predisposes patients to unique and longstanding haemodynamic challenges, including elevated systemic venous pressure and lymphatic dysfunction, which contribute to the development of PLE. PLE is a severe complication characterized by excessive loss of plasma proteins into the gastrointestinal tract, leading to hypoalbuminemia, oedema, ascites, and immunodeficiency, which can lead to frequent hospitalizations. The management is challenging and to date limited; it involves merely supportive measures such as albumin and immunoglobulin infusions, diuretics, corticosteroids, and interventional procedures targeting anatomical obstructions.^6–9^ However, these treatments do not always provide and ensure durable improvement. SGLT2i use in patients with FC failure and PLE is currently a new field of investigation.^4^

The MR lymphangiography is a useful diagnostic to identify and evaluate treatment targets to limit, at least temporarily, loss of protein and albumin in patients with lymphatic permeability issues, including patients with failing Fontan. The MR lymphangiography is non-invasive, radiation-free, expensive, and long diagnostic, which involves the coordination of multiple teams (interventional team, radiologist, and anaesthetists).

In our patient, it allowed to identify the exact area of lymphatic congestion and leakage into the duodenal lumen. Amongst all the palliative medical and invasive treatments offered to our patient, the introduction of SGLT-2i was associated with a significant and rapid clinical improvement, marked reduction in ascites, stabilization of serum protein and albumin levels, and no further hospitalizations over an extended follow-up of 18 months. Importantly, renal function and glomerular filtration rate (GFR) remained stable throughout the treatment, and echocardiogram and CPET parameters did not worsen.

The mechanisms through which SGLT-2 inhibitors may act in this setting remain not completely clarified. Their diuretic and natriuretic properties could possibly reduce venous congestion and systemic venous pressures, potentially favouring improved lymphatic function; likewise, their anti-inflammatory and metabolic effects might play a supportive role.^10^ These remain hypotheses and should be regarded as exploratory rather than explanatory.

Moreover, the relatively short interval of ∼1 month, between the stenting in the stent procedure of the Fontan conduit and the drug initiation, makes it difficult to exclude the possibility that delayed haemodynamic benefits from the invasive procedure contributed to the observed effect and clinical course. This potential interaction limits the strength of the interpretation and suggests that the findings should be interpreted with caution.

Although this is a single case report, it suggests that SGLT2 inhibitors could potentially serve as an adjunctive therapy in managing Fontan failure complicated by PLE, particularly when conventional treatments are insufficient. Further studies, ideally multicenter, are needed to assess safety, efficacy, and the mechanisms underlying any observed clinical improvements.

## Conclusions

SGLT2i use may represent a treatment option in patients with failing FC complicated by PLE. Lymphoscintigraphy played a critical role in diagnosing and quantifying lymphatic dysfunction. Multicenter studies are warranted to better understand the efficacy, safety, and mechanism of SGLT-2i in this cohort of patients.

## Lead author biography


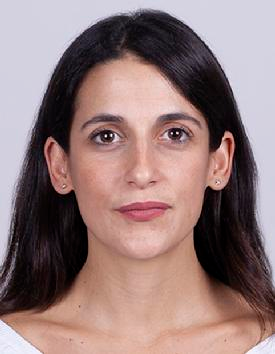


Emanuela C. D'Angelo, MD, PhDGuy's and St Thomas' NHS Foundation Trust, London, UKAdult Congenital Heart Disease Unit, Bambino Gesù Children's Hospital, IRCCS, Rome, Italy.

## Data Availability

The data underlying this article are available in the article and in its online Supplementary material.

## Notes

**Consent:** The patient has given informed consent for the publication of his clinical case.

### Fundings

This work was supported by the Italian Ministry of Health with ‘Current Research funds’.
